# Bis(2,2′-bipyridine *N*,*N*′-dioxide)bis­(tri­cyano­methanido)manganese(II)

**DOI:** 10.1107/S1600536809013828

**Published:** 2009-04-22

**Authors:** Jun Luo, Feng Yang, Li-Juan Qiu, Xin-Xia Wang, Bao-Shu Liu

**Affiliations:** a School of Pharmacy, Second Military Medical University, Shanghai 200433, People’s Republic of China; b Department of Pharmacy, Eastern Hepatobiliary Surgery Hospital, Second Military Medical University, Shanghai 200438, People’s Republic of China

## Abstract

In the title complex, [Mn(C_4_N_3_)_2_(C_10_H_8_N_2_O_2_)_2_], the Mn^II^ atom lies on an inversion center and is coordinated by two 2,2′-bipyridine *N*,*N*′-dioxide (dpdo) mol­ecules and two tricyano­methanide (tcm) ligands to form a distorted octa­hedral geometry. Weak inter­molecular C—H⋯O or C—H⋯N hydrogen bonds, involving either the O atom of the dpdo mol­ecule and the pyridyl H atom, or the N atom of the tcm anion and the pyridyl H atom, result in the formation of a three-dimensional network structure.

## Related literature

For studies of other coordination polymers constructed with tcm, exhibiting a variety of structures, see: Batten & Murray (2003 ▶); Miller & Manson (2001 ▶); Feyerherm *et al.* (2003 ▶, 2004 ▶); Abrahams *et al.* (2003 ▶); Manson *et al.* (1998 ▶, 2000 ▶); Hoshino *et al.* (1999 ▶); Batten *et al.* (1998 ▶, 1999 ▶, 2000 ▶); Manson & Schlueter (2004 ▶). For work on manganese–nitroxide complexes, see: Liu *et al.* (2001 ▶).
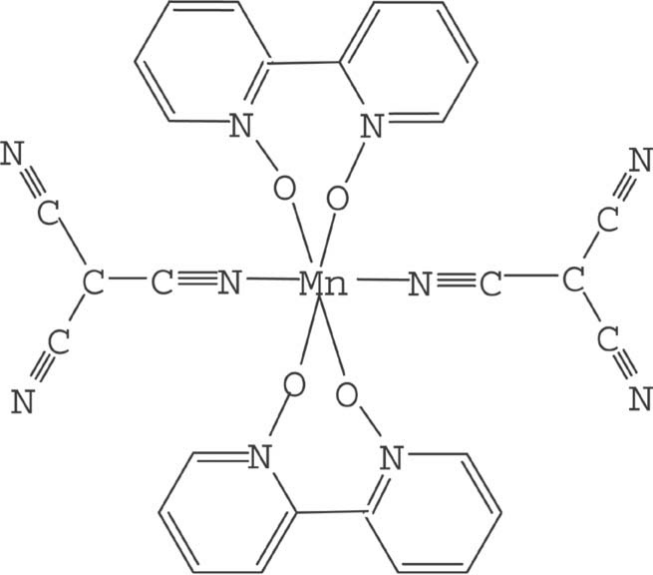


## Experimental

### 

#### Crystal data

[Mn(C_4_N_3_)_2_(C_10_H_8_N_2_O_2_)_2_]
*M* *_r_* = 611.45
Monoclinic, 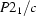

*a* = 11.514 (4) Å
*b* = 16.101 (5) Å
*c* = 7.143 (2) Å
β = 94.375 (4)°
*V* = 1320.4 (7) Å^3^
*Z* = 2
Mo *K*α radiation
μ = 0.56 mm^−1^
*T* = 293 K
0.20 × 0.16 × 0.10 mm

#### Data collection

Bruker SMART CCD area-detector diffractometer
Absorption correction: multi-scan (*SADABS*; Sheldrick, 1996 ▶) *T*_min_ = 0.893, *T*_max_ = 0.937
6266 measured reflections
2834 independent reflections
1969 reflections with *I* > 2σ(*I*)
*R*_int_ = 0.034

#### Refinement

*R*[*F* ^2^ > 2σ(*F* ^2^)] = 0.034
*wR*(*F* ^2^) = 0.071
*S* = 0.90
2834 reflections
196 parameters
H-atom parameters constrained
Δρ_max_ = 0.28 e Å^−3^
Δρ_min_ = −0.35 e Å^−3^

### 

Data collection: *SMART* (Bruker, 2000 ▶); cell refinement: *SAINT-Plus* (Bruker, 2000 ▶); data reduction: *SAINT-Plus*; program(s) used to solve structure: *SHELXS97* (Sheldrick, 2008 ▶); program(s) used to refine structure: *SHELXL97* (Sheldrick, 2008 ▶); molecular graphics: *PLATON* (Spek, 2009 ▶); software used to prepare material for publication: *SHELXTL* (Sheldrick, 2008 ▶).

## Supplementary Material

Crystal structure: contains datablocks global, I. DOI: 10.1107/S1600536809013828/dn2445sup1.cif

Structure factors: contains datablocks I. DOI: 10.1107/S1600536809013828/dn2445Isup2.hkl

Additional supplementary materials:  crystallographic information; 3D view; checkCIF report

## Figures and Tables

**Table 1:** Hydrogen-bond geometry (Å, °)

*D*—H⋯*A*	*D*—H	H⋯*A*	*D*⋯*A*	*D*—H⋯*A*
C7—H7⋯O1^i^	0.93	2.43	3.350 (2)	171
C10—H10⋯N4^ii^	0.93	2.53	3.212 (3)	130

Symmetry codes: (i) 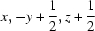; (ii) 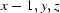.

**Fig. 1.**
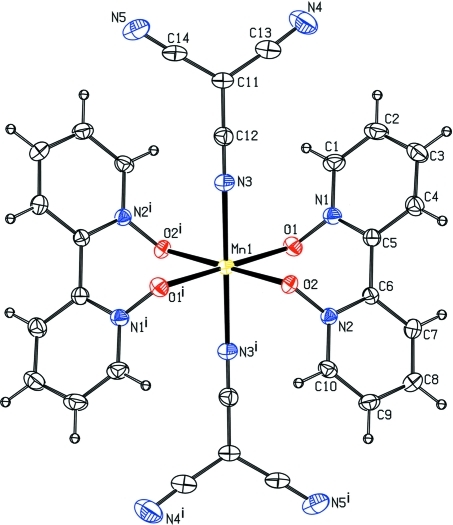
A view of the mononuclear structure in (I), showing the atom-labeling scheme. Displacement ellipsoids are drawn at the 30% probability level. [Symmetry code: (i) -x + 1, -y + 1, -z + 1.]

**Fig. 2.**
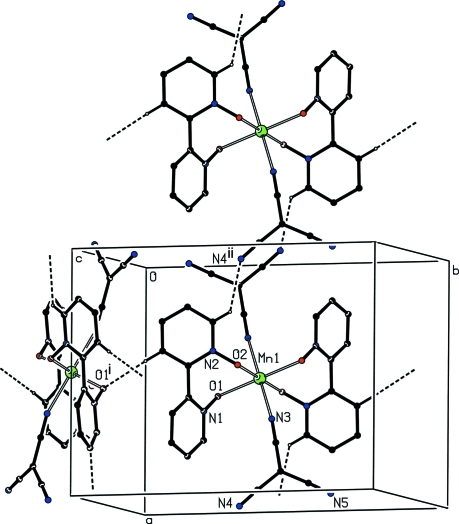
Partial packing view showing the formation of the C—H···O and C—H···N hydrogen-bond interactions. H atoms not involved in hydrogen bonding have been omitted for clarity. [Symmetry codes: (i) x, -y + 1/2, z + 1/2; (ii) x - 1, y, z.]

## supplementary crystallographic information

### Comment

Coordination polymers constructed by tricyanomethanide (tcm) have attracted considerable interest due to their diverse structures and fascinating magnetic properties (Batten & Murray, 2003; Miller & Manson, 2001; Feyerherm *et al.*, 2003). Notably, except a doubly interpenetrated (6,3) sheet was observed in Ag(tcm)_2_ (Abrahams *et al.*, 2003), most binary tcm complexes display a rutile-like structure (Manson *et al.*, 2000, 1998; Hoshino *et al.*, 1999; Feyerherm *et al.*, 2004). To gain insight into the influence of the coligands on the structures and magnetic properties of tcm complexes, some coligands such as hexamethylenetetramine, 4,4-bipyridyl, 1,2-bi(4-pyridyl)ethane were introduced to the binary tcm systems. Among the Cu^I^ or Cd^II^ tcm complexes with these coligands, numerous structure types range from doubly interpenetrated (4,4) sheet to three-dimensional rutile networks were observed (Batten *et al.*, 2000, 1998). By contrast, modification of the Mn^II^–tcm binary system with 4,4-bipyridyl as coligands leads to the formation of a one-dimensional chain-like structure (Manson & Schlueter, 2004). On the other hand, 2,2'-dipyridyl *N*,*N*'-dioxide (dpdo) is a novel coligand and has two potential oxygen donor atoms. However, no tcm complexes with dpdo as coligand have ever been reported. During our systematic investigation of the nature of dpdo coligand on the structures and properties of tcm complexes, we obtained a new tcm complex with dpdo as coligand, we herein report the synthesis and crystal structure of the new tricyanomethanide complex Mn(dpdo)_2_(C_4_N_3_)_2_ (I).

The Mn atom which lies on an inversion center displays an octahedral geometry in which the equatorial plane is formed by four O atoms (O1, O2, O1^i^, O2^i^) of the dpdo molecules, and the apical sites are occupied by two N atom (N3, N3^i^) of the tcm ligands (Fig. 1). The Mn—O(dpdo) distances are in the range from 2.1290 (13) Å to 2.1780 (13) Å, these value are comparable to the corresponding distances in manganese–nitroxide complexes (Liu *et al.*, 2001). The Mn—N(tcm) distances are from 2.2336 (17) Å to 2.2337 (17) Å, and the data are similar to the corresponding distances observed in manganese tcm complex (Batten *et al.*, 1999). Each tricyanomethanide moiety is almost planar. Bond distances and bond angles within the anions are in good agreement with those found in other tricyanomethanide complexes (Hoshino *et al.*, 1999; Batten *et al.*, 1999).

Weak intermolecular C—H···O or C—H···N hydrogen bonds involving either the O atom of the dpdo molecule and the pyridyl H atom or the N atom of the tcm anion and the pyridyl H atom, result in the formation of a three-dimensional network structure (Table 1, Fig. 2).

### Experimental

A 5 ml warm ethanol solution of 2,2'-dipyridyl *N*,*N*'-dioxide (0.10 mmol, 18.82 mg) and a 2 ml aqueous colorless solution of manganese nitrate (0.10 mmol, 25.10 mg) were mixed and stirred for 5 min, the mixed solution was yellow. To the mixture was added a 3 ml ethanol–water solution (EtOH:H_2_O = 2:1, v/v) of potassium tricyanomethanide (0.20 mmol, 25.83 mg). After stirred for another 5 min, the yellow solution was filtered and the filtrate was slowly evaporated in air. After two week, yellow block crystals of I were isolated in 34% yield. Anal: Calculated for C28H16MnN10O4: C 55.00%, H 2.64%, N 22.91%. Found C 55.16%, H 2.73%, N 23.03%.

### Refinement

In I the dpdo H atoms were placed in geometrically idealized positions and constrained to ride on their parent atoms with C—H distances of 0.93 Å and Uiso = 1.2U_eq_(C).

### Crystal data

**Table:**

[Mn(C_4_N_3_)_2_(C_10_H_8_N_2_O_2_)_2_]	*F*(000) = 622
*M**_r_* = 611.45	*D*_x_ = 1.538 Mg m^−^^3^
Monoclinic, *P*2_1_/*c*	Mo *K*α radiation, λ = 0.71073 Å
Hall symbol: -P 2ybc	Cell parameters from 954 reflections
*a* = 11.514 (4) Å	θ = 3.1–24.8°
*b* = 16.101 (5) Å	µ = 0.56 mm^−^^1^
*c* = 7.143 (2) Å	*T* = 293 K
β = 94.375 (4)°	Block, yellow
*V* = 1320.4 (7) Å^3^	0.20 × 0.16 × 0.10 mm
*Z* = 2	

### Data collection

**Table:**

Bruker SMART CCD area-detector diffractometer	2834 independent reflections
Radiation source: fine-focus sealed tube	1969 reflections with *I* > 2σ(*I*)
graphite	*R*_int_ = 0.034
φ and ω scans	θ_max_ = 27.0°, θ_min_ = 1.8°
Absorption correction: multi-scan (*SADABS*; Sheldrick, 1996)	*h* = −14→14
*T*_min_ = 0.893, *T*_max_ = 0.937	*k* = −20→20
6266 measured reflections	*l* = −5→8

### Refinement

**Table:**

Refinement on *F*^2^	Primary atom site location: structure-invariant direct methods
Least-squares matrix: full	Secondary atom site location: difference Fourier map
*R*[*F*^2^ > 2σ(*F*^2^)] = 0.034	Hydrogen site location: inferred from neighbouring sites
*wR*(*F*^2^) = 0.071	H-atom parameters constrained
*S* = 0.90	*w* = 1/[σ^2^(*F*_o_^2^) + (0.0269*P*)^2^] where *P* = (*F*_o_^2^ + 2*F*_c_^2^)/3
2834 reflections	(Δ/σ)_max_ < 0.001
196 parameters	Δρ_max_ = 0.28 e Å^−^^3^
0 restraints	Δρ_min_ = −0.34 e Å^−^^3^

### Special details

**Table:**

Geometry. All esds (except the esd in the dihedral angle between two l.s. planes) are estimated using the full covariance matrix. The cell esds are taken into account individually in the estimation of esds in distances, angles and torsion angles; correlations between esds in cell parameters are only used when they are defined by crystal symmetry. An approximate (isotropic) treatment of cell esds is used for estimating esds involving l.s. planes.
Refinement. Refinement of *F*^2^ against ALL reflections. The weighted *R*-factor *wR* and goodness of fit *S* are based on *F*^2^, conventional *R*-factors *R* are based on *F*, with *F* set to zero for negative *F*^2^. The threshold expression of *F*^2^ > σ(*F*^2^) is used only for calculating *R*-factors(gt) *etc*. and is not relevant to the choice of reflections for refinement. *R*-factors based on *F*^2^ are statistically about twice as large as those based on *F*, and *R*- factors based on ALL data will be even larger.

### Fractional atomic coordinates and isotropic or equivalent isotropic displacement parameters (Å^2^)

**Table:**

	*x*	*y*	*z*	*U*_iso_*/*U*_eq_	
Mn1	0.5000	0.5000	0.5000	0.02993 (12)	
N1	0.64563 (12)	0.36945 (9)	0.7192 (2)	0.0332 (4)	
N2	0.41397 (12)	0.43113 (9)	0.8531 (2)	0.0295 (3)	
N3	0.67180 (13)	0.55973 (10)	0.5796 (2)	0.0455 (4)	
N4	1.03850 (17)	0.50470 (13)	0.7829 (3)	0.0790 (7)	
N5	0.96600 (16)	0.72092 (13)	0.4362 (3)	0.0662 (6)	
O1	0.57417 (10)	0.37808 (7)	0.56602 (18)	0.0372 (3)	
O2	0.46743 (10)	0.49721 (7)	0.78936 (16)	0.0324 (3)	
C1	0.76219 (16)	0.36510 (12)	0.7024 (3)	0.0438 (5)	
H1	0.7909	0.3727	0.5854	0.053*	
C2	0.83767 (17)	0.34971 (12)	0.8549 (4)	0.0508 (6)	
H2	0.9172	0.3464	0.8412	0.061*	
C3	0.79652 (17)	0.33917 (12)	1.0283 (3)	0.0476 (5)	
H3	0.8473	0.3280	1.1327	0.057*	
C4	0.67888 (16)	0.34541 (11)	1.0448 (3)	0.0413 (5)	
H4	0.6499	0.3388	1.1619	0.050*	
C5	0.60310 (15)	0.36128 (10)	0.8902 (3)	0.0326 (4)	
C6	0.47602 (15)	0.36233 (11)	0.9049 (3)	0.0306 (4)	
C7	0.42058 (17)	0.29644 (12)	0.9818 (3)	0.0407 (5)	
H7	0.4629	0.2493	1.0191	0.049*	
C8	0.30314 (17)	0.29968 (13)	1.0040 (3)	0.0456 (5)	
H8	0.2659	0.2555	1.0580	0.055*	
C9	0.24191 (16)	0.36896 (12)	0.9453 (3)	0.0416 (5)	
H9	0.1621	0.3716	0.9566	0.050*	
C10	0.29796 (15)	0.43425 (12)	0.8701 (3)	0.0356 (5)	
H10	0.2560	0.4812	0.8304	0.043*	
C11	0.88681 (16)	0.59756 (12)	0.6110 (3)	0.0400 (5)	
C12	0.76883 (17)	0.57701 (12)	0.5935 (3)	0.0375 (5)	
C13	0.96832 (18)	0.54529 (14)	0.7082 (3)	0.0510 (6)	
C14	0.92869 (16)	0.66597 (14)	0.5135 (3)	0.0457 (6)	

### Atomic displacement parameters (Å^2^)

**Table:**

	*U*^11^	*U*^22^	*U*^33^	*U*^12^	*U*^13^	*U*^23^
Mn1	0.0255 (2)	0.0319 (2)	0.0322 (2)	−0.00346 (17)	0.00101 (17)	0.00237 (19)
N1	0.0290 (8)	0.0270 (8)	0.0434 (10)	0.0017 (7)	0.0015 (8)	−0.0001 (7)
N2	0.0299 (8)	0.0302 (9)	0.0283 (8)	−0.0011 (7)	0.0022 (7)	0.0002 (7)
N3	0.0343 (9)	0.0523 (11)	0.0488 (11)	−0.0114 (8)	−0.0041 (8)	0.0086 (9)
N4	0.0531 (12)	0.0831 (16)	0.0977 (18)	0.0124 (12)	−0.0146 (12)	0.0026 (14)
N5	0.0501 (12)	0.0682 (14)	0.0823 (16)	−0.0136 (10)	0.0188 (11)	0.0018 (12)
O1	0.0382 (7)	0.0355 (7)	0.0373 (8)	0.0019 (6)	−0.0017 (6)	−0.0017 (6)
O2	0.0357 (7)	0.0273 (7)	0.0346 (7)	−0.0034 (6)	0.0051 (6)	0.0036 (6)
C1	0.0307 (11)	0.0434 (12)	0.0586 (14)	0.0050 (9)	0.0128 (11)	0.0003 (11)
C2	0.0276 (10)	0.0438 (13)	0.0807 (18)	0.0037 (9)	0.0020 (12)	0.0016 (12)
C3	0.0373 (12)	0.0405 (12)	0.0627 (15)	0.0046 (9)	−0.0111 (11)	0.0025 (11)
C4	0.0406 (12)	0.0356 (11)	0.0469 (13)	0.0032 (9)	−0.0019 (10)	0.0042 (10)
C5	0.0317 (10)	0.0250 (10)	0.0410 (12)	0.0016 (8)	0.0020 (9)	0.0013 (9)
C6	0.0296 (9)	0.0294 (10)	0.0324 (11)	0.0008 (8)	0.0002 (8)	0.0029 (8)
C7	0.0409 (11)	0.0327 (11)	0.0481 (13)	−0.0008 (9)	0.0019 (10)	0.0098 (10)
C8	0.0415 (12)	0.0456 (13)	0.0502 (13)	−0.0103 (10)	0.0081 (11)	0.0087 (11)
C9	0.0291 (10)	0.0533 (13)	0.0430 (12)	−0.0048 (10)	0.0074 (9)	−0.0001 (11)
C10	0.0288 (10)	0.0435 (12)	0.0346 (11)	0.0061 (9)	0.0018 (9)	−0.0014 (9)
C11	0.0265 (10)	0.0460 (12)	0.0473 (13)	−0.0047 (9)	0.0019 (9)	−0.0035 (10)
C12	0.0376 (11)	0.0382 (12)	0.0360 (12)	−0.0024 (9)	−0.0011 (9)	−0.0021 (9)
C13	0.0349 (12)	0.0564 (14)	0.0612 (16)	−0.0020 (11)	0.0008 (11)	−0.0109 (12)
C14	0.0249 (10)	0.0566 (15)	0.0558 (15)	−0.0023 (10)	0.0056 (10)	−0.0099 (12)

### Geometric parameters (Å, °)

**Table:**

Mn1—O2	2.1291 (13)	C2—H2	0.9300
Mn1—O2^i^	2.1291 (13)	C3—C4	1.372 (3)
Mn1—O1^i^	2.1780 (13)	C3—H3	0.9300
Mn1—O1	2.1780 (13)	C4—C5	1.378 (3)
Mn1—N3^i^	2.2337 (17)	C4—H4	0.9300
Mn1—N3	2.2337 (17)	C5—C6	1.475 (2)
N1—O1	1.3252 (19)	C6—C7	1.374 (2)
N1—C5	1.356 (2)	C7—C8	1.374 (3)
N1—C1	1.358 (2)	C7—H7	0.9300
N2—O2	1.3269 (16)	C8—C9	1.368 (3)
N2—C10	1.351 (2)	C8—H8	0.9300
N2—C6	1.354 (2)	C9—C10	1.365 (2)
N3—C12	1.148 (2)	C9—H9	0.9300
N4—C13	1.140 (3)	C10—H10	0.9300
N5—C14	1.144 (2)	C11—C12	1.394 (2)
C1—C2	1.363 (3)	C11—C13	1.404 (3)
C1—H1	0.9300	C11—C14	1.408 (3)
C2—C3	1.370 (3)		
			
O2—Mn1—O2^i^	180.0	C2—C3—C4	118.7 (2)
O2—Mn1—O1^i^	97.70 (4)	C2—C3—H3	120.6
O2^i^—Mn1—O1^i^	82.30 (4)	C4—C3—H3	120.6
O2—Mn1—O1	82.30 (4)	C3—C4—C5	120.9 (2)
O2^i^—Mn1—O1	97.70 (4)	C3—C4—H4	119.6
O1^i^—Mn1—O1	180.0	C5—C4—H4	119.6
O2—Mn1—N3^i^	91.14 (5)	N1—C5—C4	119.32 (16)
O2^i^—Mn1—N3^i^	88.86 (5)	N1—C5—C6	119.42 (17)
O1^i^—Mn1—N3^i^	90.45 (6)	C4—C5—C6	121.05 (18)
O1—Mn1—N3^i^	89.55 (6)	N2—C6—C7	119.30 (16)
O2—Mn1—N3	88.86 (5)	N2—C6—C5	119.70 (15)
O2^i^—Mn1—N3	91.14 (5)	C7—C6—C5	120.87 (17)
O1^i^—Mn1—N3	89.55 (6)	C6—C7—C8	120.52 (18)
O1—Mn1—N3	90.45 (6)	C6—C7—H7	119.7
N3^i^—Mn1—N3	180.00 (8)	C8—C7—H7	119.7
O1—N1—C5	120.64 (14)	C9—C8—C7	118.95 (18)
O1—N1—C1	119.20 (17)	C9—C8—H8	120.5
C5—N1—C1	120.09 (17)	C7—C8—H8	120.5
O2—N2—C10	119.25 (14)	C10—C9—C8	120.03 (18)
O2—N2—C6	120.05 (14)	C10—C9—H9	120.0
C10—N2—C6	120.68 (15)	C8—C9—H9	120.0
C12—N3—Mn1	164.42 (17)	N2—C10—C9	120.47 (18)
N1—O1—Mn1	118.80 (10)	N2—C10—H10	119.8
N2—O2—Mn1	118.06 (10)	C9—C10—H10	119.8
N1—C1—C2	120.8 (2)	C12—C11—C13	120.78 (19)
N1—C1—H1	119.6	C12—C11—C14	120.63 (18)
C2—C1—H1	119.6	C13—C11—C14	118.19 (17)
C1—C2—C3	120.09 (19)	N3—C12—C11	179.7 (2)
C1—C2—H2	120.0	N4—C13—C11	176.8 (2)
C3—C2—H2	120.0	N5—C14—C11	178.0 (2)

Symmetry codes: (i) −*x*+1, −*y*+1, −*z*+1.

### Hydrogen-bond geometry (Å, °)

**Table:**

*D*—H···*A*	*D*—H	H···*A*	*D*···*A*	*D*—H···*A*
C7—H7···O1^ii^	0.93	2.43	3.350 (2)	171
C10—H10···N4^iii^	0.93	2.53	3.212 (3)	130

Symmetry codes: (ii) *x*, −*y*+1/2, *z*+1/2; (iii) *x*−1, *y*, *z*.
